# Onion peel extracts ameliorate hyperglycemia and insulin resistance in high fat diet/streptozotocin-induced diabetic rats

**DOI:** 10.1186/1743-7075-8-18

**Published:** 2011-03-28

**Authors:** Ji Young Jung, Yeni Lim, Min Sun Moon, Ji Yeon Kim, Oran Kwon

**Affiliations:** 1 Department of Nutritional Science and Food Management, Ewha Womans University, 11-1 Daehyeon-dong, Seodeamun-gu, Seoul 120-750, Republic of Korea

**Keywords:** Onion Peel Extract, Quercetin, Type 2 Diabetes, Streptozotocin, Antioxidant

## Abstract

**Background:**

Quercetin derivatives in onions have been regarded as the most important flavonoids to improve diabetic status in cells and animal models. The present study was aimed to examine the hypoglycemic and insulin-sensitizing capacity of onion peel extract (OPE) containing high quercetin in high fat diet/streptozotocin-induced diabetic rats and to elucidate the mechanism of its insulin-sensitizing effect.

**Methods:**

Male Sprague-Dawley rats were fed the AIN-93G diet modified to contain 41.2% fat and intraperitoneally injected with a single dose of streptozotocin (40 mg/kg body weight). One week after injection, the rats with fasting blood glucose levels above 126 mg/dL were randomly divided into 4 groups to treat with high fat diet containing 0 (diabetic control), 0.5, or 1% of OPE or 0.1% quercetin (quercetin equivalent to 1% of OPE) for 8 weeks. To investigate the mechanism for the effects of OPE, we examined biochemical parameters (insulin sensitivity and oxidative stresses) and protein and gene expressions (pro-inflammatory cytokines and receptors).

**Results:**

Compared to the diabetic control, hypoglycemic and insulin-sensitizing capability of 1% OPE were demonstrated by significant improvement of glucose tolerance as expressed in incremental area under the curve (*P *= 0.0148). The insulin-sensitizing effect of OPE was further supported by increased glycogen levels in liver and skeletal muscle (*P *< 0.0001 and *P *= 0.0089, respectively). Quantitative RT-PCR analysis showed increased expression of insulin receptor (*P *= 0.0408) and GLUT4 (*P *= 0.0346) in muscle tissues. The oxidative stress, as assessed by superoxide dismutase activity and malondialdehyde formation, plasma free fatty acids, and hepatic protein expressions of IL-6 were significantly reduced by 1% OPE administration (*P *= 0.0393, 0.0237, 0.0148 and 0.0025, respectively).

**Conclusion:**

OPE might improve glucose response and insulin resistance associated with type 2 diabetes by alleviating metabolic dysregulation of free fatty acids, suppressing oxidative stress, up-regulating glucose uptake at peripheral tissues, and/or down-regulating inflammatory gene expression in liver. Moreover, in most cases, OPE showed greater potency than pure quercetin equivalent. These findings provide a basis for the use of onion peel to improve insulin insensitivity in type 2 diabetes.

## Background

Type 2 diabetes mellitus (T2DM) is one of the world's most common chronic diseases as changing lifestyles lead to reduced physical activity and increased obesity [1]. Early phenomenon of T2DM is insulin insensitivity, which not only has negative metabolic consequences [2-5] but also contributes subsequent pancreas β-cell exhaustion, resulting in the onset of clinical hyperglycemia [6]. Thus, understanding the regulation of the insulin response and identifying the related mechanisms are important to early treatment and prevention of T2DM. Several hypotheses have been proposed to explain the pathogenesis of T2DM, and during last decades, much attention has been given to the lipid toxicity and low-grade inflammation as major causes on insulin insensitivity [7,8].

A number of ways to improve insulin sensitivity have been proposed, because early treatment and prevention play a pivotal role in reducing the population burden of diabetes. Lifestyle changes such as losing weight, exercising, and watching the diet are often recommended, but have been difficult to maintain over a long term. Benefits of pharmaceutical factors to treat the disease aggressively early have been recommended, but medications may have unwanted side effects. Thus, there has been a growing interest in herbal remedies that can be introduced into the general population with the least side effects and the maximal preventive outcome [9]. In this context, flavonoids naturally occurring in plant foods would be desirable options. Many studies have shown that diabetes can be delayed or prevented by intervention with dietary flavonoids. Quercetin is one of the most common flavonoids in foods and has been reported to improve diabetic status by decreasing oxidative stress [10-12] or by reducing the disturbance of hepatic gene expressions [13]. However, most of the studies are generally carried out using highly purified quercetin rather than food extracts enriched in quercetin.

Onion bulbs have been recognized as the richest source of dietary flavonoids. At least 25 different flavonoids have been characterized and quercetin and its glycosides are the most important ones [14]. Especially higher concentrations of quercetin occur in the outer dry layers of onion bulb [15]. In the previous study, our team showed that outer dry layers of onion bulb have strong antioxidant activity and proposed quercetin as the major component responsible for this activity [16]. Although there are a few studies presented antidiabetic effects of onion skin extract *in vivo *[17,18], more evidence is needed to support its insulin-sensitizing capabilities. Therefore the present study was performed to evaluate the effectiveness of OPE in modulating hyperglycemia and insulin-insensitivity by using a high fat diet (HFD)/streptozotocin (STZ)-induced diabetic rat model. The effect of OPE on the plasma concentration of FFAs, the biomarkers of oxidative stress and inflammation in liver, the insulin receptor (INSR) and glucose transporter type 4 (GLUT4) expressions in skeletal muscle were also investigated.

## Materials and methods

### Preparation of OPE

The OPE was kindly provided by the Center for Changnyeong Onion Bioindustry (Changwon, Korea). Briefly, outer dry layers of onion bulbs (*Allium cepa *L.) were extracted with 60% ethanol adjusted to pH 5.5 at 50°C for 3 hours. The extract was concentrated and then freeze-dried. The amount of total polyphenol and quercetin were 618.10 ± 14.51 mg/g and 101.28 ± 6.95 mg/g as determined by methods of Folin-Ciocalteu [19] and Hertog *et al. *[20], respectively. Pure quercetin was purchased from Wako Pure Chemical Industries, Ltd. (Osaka, Japan).

### Animals, induction of diabetics, and diets

Male Sprague-Dawley rats aged 8 weeks were purchased from Orient Bio Inc. (Seoul, Korea). The rats were housed at a temperature of 23 ± 1°C with 12/12 h light/dark cycles and 45 ± 5% humidity with access to water and chow diet for a week prior to the experiment. The experimental protocols were approved by the Institutional Animal Care and Use Committee (IACUC) of the Ewha Womans University. For the experiments, a diabetic state was induced by feeding AIN 93G diet modified to contain 41.2% fat (HFD) for 2 weeks, followed by a single intraperitoneal injection of STZ (Sigma Chemical Co., St. Louis, MO, USA) at a low dose (40 mg/kg body weight, dissolved in 0.05 M citrate buffer, pH 4.5, immediately before use). One week after injection, fasting blood glucose (FBG) levels were determined from tail blood using an Accu-Check (Roche Diagnostics, Manheim, Germany). The rats with FBG levels above 126 mg/dL were randomly divided into 4 groups (n = 7 for each group): one group was fed with a HFD only (diabetic control group), two groups with a HFD containing 0.5 or 1% of OPE respectively, and the other group with a HFD containing 0.1% of quercetin (matched with the 1% of OPE-treated group) for 8 weeks. The rats consumed diet and tap water *ad libitum *during the experimental period. Oral glucose tolerance tests (OGTTs) were performed from the tail vein at the last week of the experimental period. The rats were sacrificed under anesthesia and blood, liver, and skeletal muscle was immediately collected.

### Oral glucose tolerance test

After overnight fasting for 12 hours, the animals were administered a glucose (1 g/kg of body weight) dissolved in water by *gavage*. Blood glucose concentrations were determined from the tail vein with an Accu-check at 0, 30, 60, 90, and 120 min. The incremental area under the curve (IAUC) was calculated using the method of Thomas MS Wolever *et al. *[21].

### Measurement of plasma insulin and free fatty acid

Plasma insulin and FFAs were measured using commercial kits (Rat insulin ELISA kit, Mercodia, Uppsala, Sweden; FFA quantification kit, Biovision, Mountain view, CA, USA). All procedures were performed in accordance with the manufacturer's instructions. Homeostasis model assessment-insulin resistance (HOMA-IR) was calculated to measure the insulin sensitivity of the rats fed the experimental diets by the following formula [22,23]: [Fasting plasma insulin (μg/L) × Fasting blood glucose (mg/dL)]/22.5.

### Measurements of glycogen synthesis

Glycogen in liver and skeletal muscle was determined using the method described by LO *et al *[24]. Briefly, samples were homogenized in KOH solution and incubated in boiling water for 30 min. After addition of 90% ethanol, they were incubated overnight at 4°C, followed by the centrifugation at 1,000 g for 30 min at 4°C (H50A-8 centrifuge, Hanil, Seoul, Korea). Water, 5% phenol reagent, and H_2_SO_4 _solution were added to standard and samples. Bovine liver glycogen (Sigma Chemical Co., St. Louis, MO, USA) was used as a standard. Absorbance at 490nm was determined using a spectrophotometer (Genesys 10UV, Thermo Electron Co., Madison, WI, USA).

### Measurements of liver markers of oxidative stress

Liver malondialdehyde (MDA) and superoxide dismutase (SOD) activity were measured using a commercial kit purchased from Cayman Chemical (Ann Arbor, MI, USA) and Dojindo Lab (Kumamoto, Japan) respectively. For MDA, liver was homogenized in 250 μl of radioimmunoprecipitation buffer containing protease inhibitor and sonicated for 15 sec at 40 V. After centrifugation at 1,600 g for 10 min at 4°C, the supernatant was collected to measure MDA concentration according to the manufacturer's instructions. For SOD activity, liver was homogenized in 10 volumes (w/v) of 50 mM phosphate - 0.25 M sucrose - 0.5 mM EDTA buffer (pH 7.4). The homogenate was centrifuged at 10,000 g for 20 min at 4°C. Five milliliters of the supernatant was ultrasonicated twice for 30 sec. Next, 2 ml of solution containing five volumes of chloroform with three volumes of ethanol was added and mixed strongly for 2 min. The mixture was centrifuged at 20,000 g for 20 min at 4°C. The final supernatant was collected and measured for SOD activity according to the manufacturer's instructions.

### Western blotting

Equal amounts of liver whole lysate were separated by SDS-polyacrylamide gel electrophoresis, transferred to polyvinylidene difluoride membranes, incubated in blocking buffer, and treated with primary antibodies: anti-tumor necrosis factor (TNF)-α (Abcam, Cambridge, UK), anti-interleukin (IL)-6 (Abcam, Cambridge, UK), and anti-α-tubulin (Cell Signaling Technology, Danvers, MA, USA). Appropriate secondary antibodies were used, and the bands were visualized using ChemiDoc XRS System and analyzed using Quantity One software (Bio-Rad, CA, USA).

### Quantitative real-time reverse transcription-polymerase chain reaction (RT-PCR) analysis

Total RNA was extracted from liver or skeletal muscle using TRIZOL reagent (Invitrogen Co., Carlsbad, CA, USA). RNA concentration and quality were measured by a BioSpec-nano (Shimadzu Corp., Tokyo, Japan). cDNA was constructed using high capacity RNA with a cDNA kit (Applied Biosystems, Foster City, CA, USA). The expression of mRNA was measured by PCR using the TaqMan method and an ABI StepOne Plus system (Applied Biosystems, Foster City, CA, USA). The primer sets for target genes in the rats were INSR [Insr; Rn01637243_m1], GLUT-4 [Slc2a4; Rn00562597_m1], TNF-α [Tnf; Rn99999017_m1], IL-6 [Il6; Rn01410330_m1], and β-actin [Actb; Rn00667869_m1]. The relative amounts of these mRNAs were normalized to the amount of β-actin.

### Statistical analysis

All results are presented as the mean ± standard error (SE). Statistical analyses were conducted by using SAS 9.2 (SAS Institute, Cary, NC, USA). Data analyses were performed by one-way analysis of variance (ANOVA) with *post hoc *Dunnet's multiple comparison test. Statistical significance was indicated by *P *< 0.05.

## Results

### Eight-week OPE administration showed insulin sensitizing effects in HFD/STZ-induced diabetic rats

**Figure 1:**
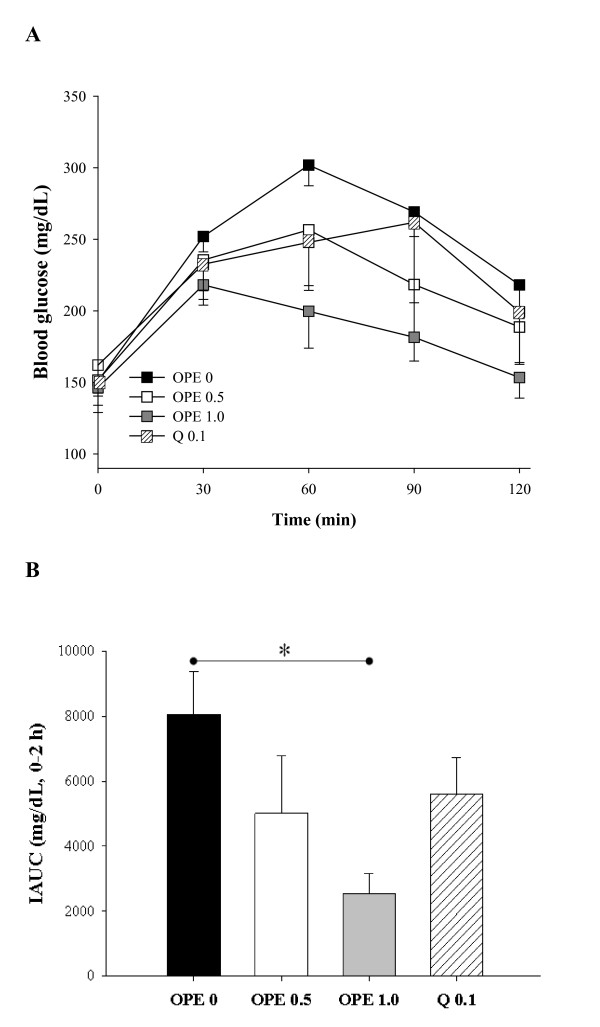
**Hypoglycemic effect of OPE or quercetin on the OGTT test**. After 8 week administration of each experimental diet in HFD/STZ induced diabetic rats, OGTTs were performed in the fasting state (A) and then IAUCs were calculated (B). Data are expressed as means ± SE (n = 7 for each group). Comparisons were done among the IAUC of each group by Dunnet's multiple comparison test. *Different from control, *P *< 0.05

**Figure 2:**
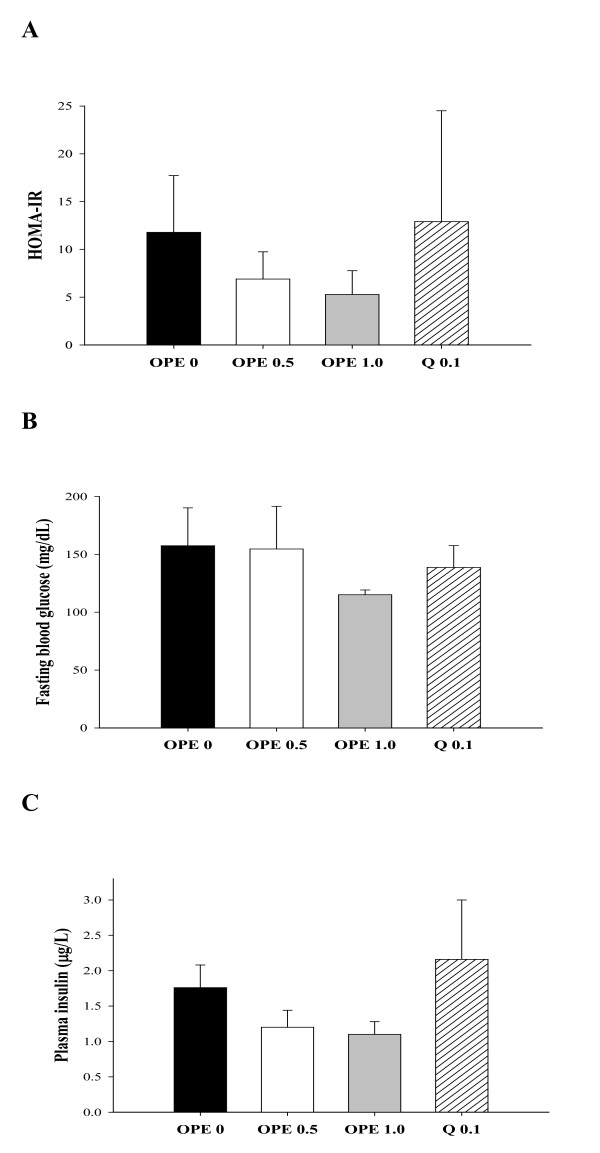
**Effects of OPE or quercetin on HOMA-IR (A), FBG (B), and insulin secretion (C) in HFD/STZ-induced diabetic rats**. Parameters were measured after 8 week administration of 0, 0.5, 1% OPE or 0.1% quercetin. Data are expressed as means ± SE (n = 7 for each group). Comparisons were done between the control group and each individual treated group by Dunnet's multiple comparison test.

To determine the insulin sensitizing effects of OPE, OGTT was performed at the last week of treatment. As compared to the diabetic control, the OPE treated groups showed consistent improvement in OGTT at all time points in a dose-dependent manner, although the differences did not reach statistical significance (Figure 1A). The difference between the diabetic control and the 1% OPE treated group was marginally significant at time points 60 min (*P *= 0.0597). As shown in Figure 1B, in comparison to the diabetic control, rats with 1% OPE administration showed significantly decreased IAUC (*P *= 0.0148), whereas the quercetin equivalent group showed no significant reduction. HOMA-IR, FBG and insulin secretion showed insignificant reduction in all treatments (Figure 2). There were also no differences in body weight, food intakes, and organ weights among groups (data not shown).

**Figure 3:**
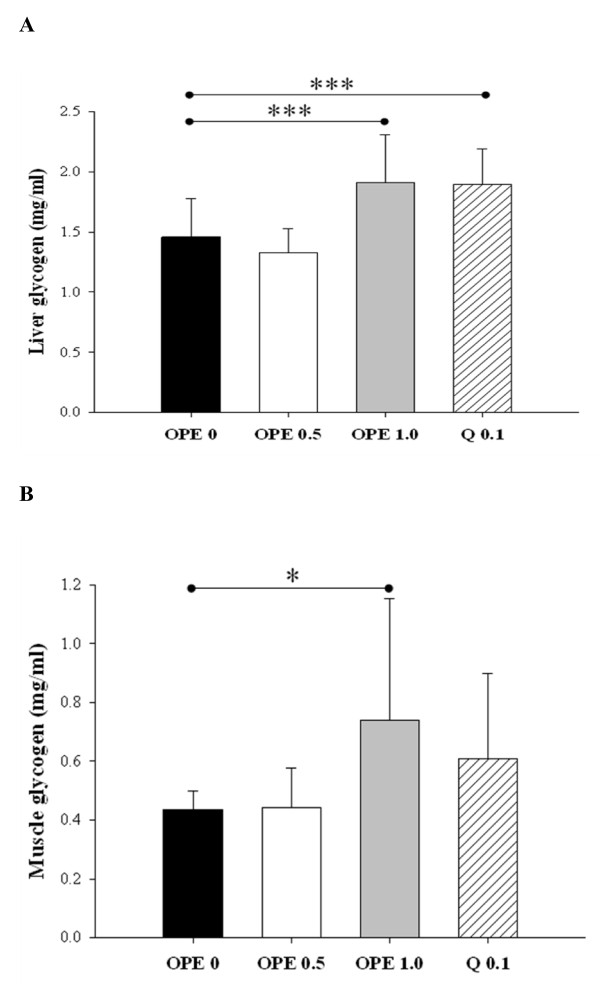
**Glycogen levels in liver (A) and skeletal muscle (B)**. Glycogen levels were measured in liver and skeletal muscle after 8 week administrations of 0, 0.5, 1% OPE or 0.1% quercetin in HFD/STZ-induced diabetic rats. Data are expressed as means ± SE (n = 7 for each group). Comparisons were done between the control group and each individual treated group by Dunnet's multiple comparison test. *Different from control, *P *< 0.05, ***different from control, *P *< 0.005

To support insulin sensitizing effect of OPE further, glucose uptake and utilization in peripheral tissues were determined. In liver, both 1% OPE and quercetin induced significant increases in glycogen levels, as compared to the diabetic control (*P *< 0.005, Figure 3A). Similar effect was shown in skeletal muscle, but the difference between the diabetic control and the quercetin equivalent was not significant (*P *= 0.2186, Figure 3B).

### Oxidative stress and metabolic dysregulation of FFAs in diabetic condition were alleviated by OPE administration

**Figure 4:**
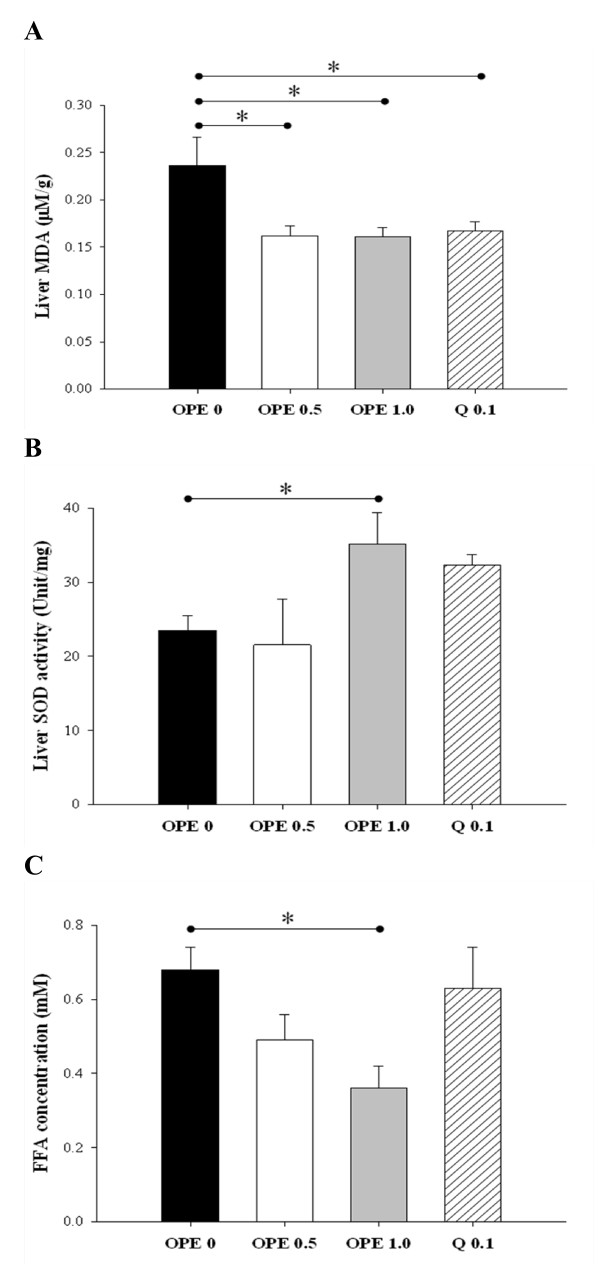
**Effect of OPE or quercetin on MDA formation (A), SOD activity (B), and plasma FFAs (C) in HFD/STZ-induced diabetic rats**. MDA formation, SOD activity and plasma FFAs were measured in liver and skeletal muscle after 8 week administrations of 0, 0.5, 1% OPE or 0.1% quercetin in HFD/STZ-induced diabetic rats. Data are expressed as means ± SE (n = 7 for each group). Comparisons were done between the control group and each individual treated group by Dunnet's multiple comparison test. * Different from control, *P *< 0.05

Oxidative stress status was assessed by measuring the MDA formation and SOD activity in liver. Compared with diabetic control, MDA formation was suppressed by quercetin as well as all 2 doses of OPE administration (*P *< 0.05, Figure 4A), whereas SOD activity was significantly increased only in the 1% OPE-treated rats (*P *= 0.0393, Figure 4B). Plasma FFA levels were decreased in a dose-dependent manner and one percent of OPE administration showed significant lower level compared with the diabetic control (*P *= 0.0148). However quercetin administration showed no change (Figure 4C).

### The OPE modified gene expressions related to inflammation and glucose uptake in the liver

**Figure 5:**
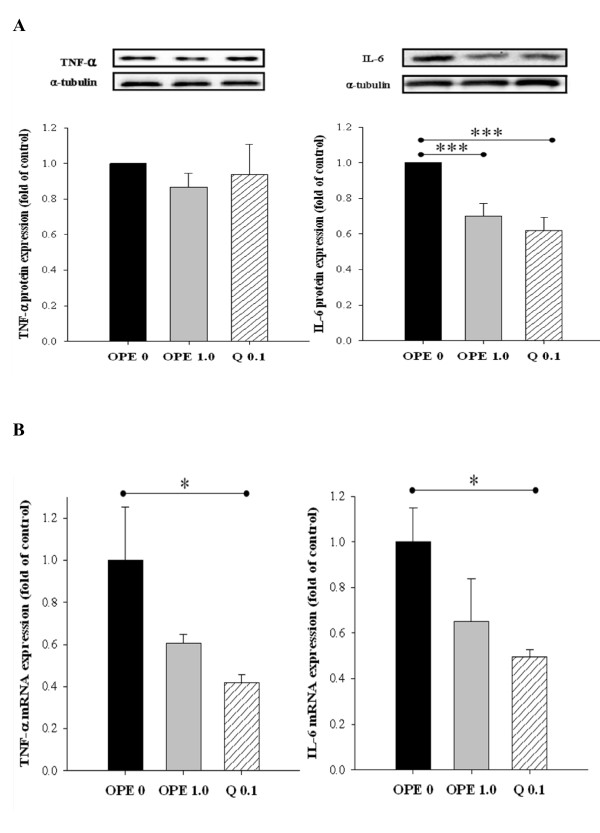
**Immunoblot analysis of protein levels (A) and gene expression levels (B) of TNF-α and IL-6 in liver of HFD/STZ-induced diabetic rats**. Levels of TNF-α protein and IL-6 protein were measured normalized against α-tublin, and expressed as fold of control. The mRNA expression levels were determined by quantitative RT-PCR, normalized against β-actin, and plotted relative to those of diabetic control rats. Data are expressed as means ± SE, (n = 7 for each group). Comparisons were done between the control group and each individual treated group by Dunnet's multiple comparison test. *Different from control, *P *< 0.05, ***different from control, *P *< 0.005

**Figure 6:**
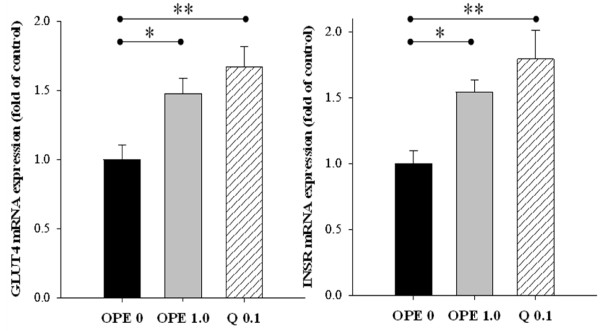
**Effects of OPE or quercetin on the mRNA expression of the INSR and GLUT 4 in skeletal muscle of HFD/STZ-induced diabetic rats**. Expression levels for INSR and GLUT4 were determined by quantitative RT-PCR, normalized against β-actin, and plotted relative to those of diabetic control rats. Data are expressed as means ± SE (n = 7 for each group). Comparisons were done between the control group and each individual treated group by Dunnet's multiple comparison test. *Different from control, *P *< 0.05, **different from control, *P *< 0.01

In comparison to the diabetic control, quercetin administration significantly suppressed mRNA expression of IL-6 gene in liver. In line with this RT-PCR result, IL-6 protein was also decreased significantly by quercetin administration. One percent of OPE administration significantly suppressed IL-6 protein in liver (*P *= 0.0025), whereas showing little suppression on mRNA expression of IL-6 gene, compared with the diabetic control. Although mRNA levels of TNF-α were decreased significantly by quercetin administration (*P *< 0.05), neither quercetin nor 1% OPE administration significantly affected TNF-α protein in liver compared with the diabetic control (Figure 5). In addition, hepatic mRNA expressions of both INSR and GLUT4 significantly increased in 1% OPE or quercetin administration compared with the diabetic control (*P *< 0.05, Figure 6).

## Discussion

This study was designed to investigate the modulating effects of OPE on hyperglycemia and insulin-insensitivity in HFD/STZ-induced diabetic rats. To induce T2DM, a single low dose of STZ at 40 mg/kg body weight was injected combined with HFD. High doses of STZ (>45 mg/kg body weight) is well known to be taken by pancreatic β-cells via GLUT2 and to induce severe damages of pancreatic β-cells, mimicking T1DM [25]. On the contrary, the combination of HFD and low doses of STZ resulted in characteristic of T2DM; HFD induces insulin resistance and low doses of intraperitoneal STZ induce moderate impairment of insulin secretion [26-30]. In this study, OGTT, insulin secretion, and peripheral glucose utilization were assessed, along with the plasma FFAs, the biomarkers relating to oxidative stress and inflammation in liver, and the expressions of GLUT4 and INSR genes in skeletal muscle. In all cases, the effects of 1% OPE were compared with those of pure quercetin equivalent.

Liver and skeletal muscle is the primary site of glucose disposal in the insulin-stimulated state [31]. The previous study demonstrated that glycogen storage was impaired in diabetic animals [32]. In the present study, 1% OPE administration showed hypoglycemic effects compared to the diabetic control, as evidenced by significant decrease in IAUC. In the meantime, glycogen levels were significantly increased in liver and skeletal muscles in response to 1% OPE administration. In addition, both 1% OPE and its quercetin equivalent induced up-regulation of INSR and GLUT4 gene expressions in skeletal muscle. The rapid insulin action to stimulate glucose uptake and metabolism in peripheral tissues is a fundamental mechanism for the maintenance of glucose homeostasis [33]. Glucose uptake is triggered by a cascade of events from insulin binding to its cell surface receptors, then the ability of insulin to increase glucose transport in muscle tissue via GLUT4 [33]. Therefore, it is proposed that OPE improves insulin sensitivity by up-regulating expressions of insulin receptor and glucose transporter as well as by promoting metabolism of glucose in peripheral tissues in diabetic rats. Interestingly, potency of 1% OPE was generally higher than that of pure quercetin equivalent. Onion bulbs contain more than 20 flavonoids other than quercetin [14]. According to our analysis, OPE was found to be composed of polyphenols at the level 60%. Of these, 16% was quercetin. Therefore, it was postulated that the higher potency of OPE might be attributed to the additive or synergistic effect of an array of phytochemicals.

Several studies suggested that impaired blood lipids are characteristic of subjects with insulin resistance, especially circulating FFAs [34-36]. These observations were supported by recent evidence that FFAs directly activate macrophage to secrete pro-inflammatory cytokines that render muscle cells insulin resistant [37]. Meanwhile the role for pro-inflammatory cytokines in regulating insulin sensitivity has been suggested by several lines of evidence. For example, subjects with T2DM exhibited higher serum levels of pro-inflammatory cytokines such as TNF-α, IL-1β, and IL-6 [38]. In addition, FFAs contribute to the increased production of reactive oxygen species and lead to the activation of stress-sensitive signaling pathways under hyperglycemic status [39]. In this study, the measurement of oxidative/inflammatory stresses was focused on the liver. Because dietary quercetin is metabolized in liver, inhibiting liver injury induced by diabetes may be particularly effective, consequently [13]. The results indicated that plasma FFA levels in diabetic rats were significantly decreased in response to OPE administration. Hepatic oxidant stress was reduced by 1% OPE, as assessed by increasing SOD activity and blocking MDA formation. Moreover, hepatic expressions of TNF-α and IL-6 were suppressed by either 1% OPE or quercetin. These results are in agreement with previous reports, in which quercetin had anti-oxidative and anti-inflammatory activities [12]. Therefore, although detailed mechanisms of action await further investigation, it is proposed that OPE leads to improved insulin sensitivity, at least in part, through enhancing lipid metabolism, reducing oxidative stress, or modulating pro-inflammatory cytokines in diabetic rats.

In conclusion, the present study has demonstrated the potency of OPE to ameliorate hyperglycemia and insulin resistance in diabetic rats. The OPE modulates glucose uptake and metabolism in peripheral tissues via INSR and GLUT4 gene expression in skeletal muscle. Furthermore, OPE lowered plasma FFA levels and suppressed oxidative and inflammatory stress in liver. These findings provide a basis for the use of onion peel and also have important implications for the prevention and early treatment of T2DM.

## Competing interests

The authors declare that they have no competing interests.

## Authors' contributions

JYK and OK designed this research; JYJ, YL, and MSM conducted research; JYJ, JYK and OK wrote the paper; and JYK and OK had primary responsibility for final content. All authors read and approved the final manuscript.
